# Nursing contribution to end-of-life care decision-making in patients with neurological diseases on an acute hospital ward: documentation of signs and symptoms

**DOI:** 10.1186/s12912-025-02897-1

**Published:** 2025-03-11

**Authors:** Gudrun Jonsdottir, Runar Vilhjalmsson, Valgerdur Sigurdardottir, Haukur Hjaltason, Marianne Elisabeth Klinke, Helga Jonsdottir

**Affiliations:** 1 Faculty of Nursing and Midwifery, School of Health Sciences, University of Iceland, Reykjavik, 101 Iceland; 2 Department of Hematology and Oncology, Landspitali, University Hospital of Iceland, Reykjavik, 101 Iceland; 3 Palliative Care Unit, Landspitali, University Hospital of Iceland, Reykjavik, 101 Iceland; 4 Faculty of Medicine, School of Health Sciences, University of Iceland, Reykjavik, 101 Iceland; 5 Department of Neurology, Landspitali, University Hospital of Iceland, Reykjavik, 101 Iceland; 6 Respiratory Section, Division of Clinical Services, Landspitali, University Hospital of Iceland, Reykjavik, 101 Iceland

**Keywords:** Palliative care, End-of-life care, Terminal care, Progressive neurological diseases, Stroke, Signs and symptoms, Hospital care, Nursing

## Abstract

**Background:**

Recognizing impending death in patients with neurological diseases presents challenges for nurses and other healthcare professionals. This study aimed to identify nursing contribution to end-of-life (EOL) care decision-making for patients with neurological diseases in an acute hospital ward and to compare signs and symptoms among subgroups of patients.

**Methods:**

In this retrospective study, we analyzed data from 209 patient health records using the Neurological End-Of-Life Care Assessment Tool to evaluate the care in the last 3 to 7 days of life. Key aspects included the need for EOL care, EOL care decision-making, signs and symptoms of imminent death, and communication with relatives. The patient records pertain to patients who died in an acute neurological ward between January 2011 and August 2020; 123 with ischemic stroke, 48 with hemorrhagic stroke, 27 with amyotrophic lateral sclerosis [ALS], and 11 with Parkinson’s disease or extrapyramidal and movement disorders [PDoed]. Both descriptive and inferential statistical analyses were performed to analyze the data.

**Results:**

Nurses identified the need for EOL care in 36% of cases and contributed to EOL decision-making as information brokers (15%), advocates (6%), and supporters (6%). They identified disease progression in 44% of the cases. The mean number of signs and symptoms in both the acute and progressive disease groups was 6.5 and ranged from 1 to 14. Patients with stroke without a documented EOL decision had more severe symptoms, including respiratory congestion (68%) and dyspnea (37%), than those with EOL decision. A higher frequency of no food intake was documented in patients with stroke receiving EOL care (*p* = 0.007) compared to those without. Among patients with ALS or PDoed, those with EOL decision showed a trend toward a higher frequency of unconsciousness or limited consciousness than those without EOL decision (*p* = 0.067). For all groups of patients, conversations with relatives occurred in 85% instances and family meetings in 93%.

**Conclusions:**

Nurses made substantial contributions to EOL care decision-making for patients with neurological diseases. To improve early identification of imminent death in patients with neurological diseases in acute hospital wards, healthcare professionals must investigate barriers contributing to delayed recognition.

**Clinical trial number:**

Not applicable

## Background

Recognizing imminent death in patients is challenging for nurses and other healthcare professionals [1, 2]. This may occur across different patient groups, including those with neurological diseases [3, 4, 5, 6, 7, 8]. To comprehend approaching death, clinicians rely on intuition and comprehensive information, including physical and psychological symptoms, tests, conversations with relatives, and observations, to comprehend approaching death [1]. This process is further complicated by organizational factors such as work pace and cultural attitudes towards death [1, 2]. Patients with neurological diseases exhibit numerous and severe signs and symptoms near the end of life (EOL) [4, 9, 10, 11, 12, 13, 14]. Some of these signs and symptoms, such as decreased consciousness, which may resemble typical reversible neurological symptoms such as those of acute stroke, may be temporary [3, 4, 8, 10, 15, 16]. This overlap accentuates the challenge of recognizing imminent death and increases the uncertainty of EOL decision-making in patients with neurological diseases [1].

EOL care marks the final step of palliative care and refers to care provided in the final stage of life, where the focus is shifted from life-sustaining treatment to comfort measures based on individual patient’s needs. This decision is the physician’s responsibility and is formally documented in the patients’ health records (PHRs) Consequently, a change in both medical and nursing treatment should be expected, such as exclusion of resuscitation and intensive care. Despite the different meanings of the terms palliative and EOL care, they are often used interchangeably. EOL care requires complex decision-making, considering the patient’s overall decline across physical, social, spiritual, and psychological dimensions [17, 18, 19]. Nurses are expected to identify signs and symptoms of imminent death, provide timely clinical assessment, and relay information to physicians and other professionals, actively contributing to EOL care decision-making [17, 18, 19]. Nurses must be supportive, advocate for patients, and influence EOL care decision-making [17] while ensuring that the care aligns with the values and preferences of patients and their relatives. In addition to providing holistic and continuous care, nurses must share vital information about signs and symptoms, medication administration, and possible outcomes with both patients who are dying and their relatives [17, 20, 21, 22, 23].

Failure to recognize imminent death may prolong suffering and hinder peaceful death [1, 2, 6, 24, 25, 26]. The clinical guidelines of the Registered Nurses Association of Ontario (RNAO) and existing literature highlight several signs that may indicate disease progression or impending death during the terminal phase of patients with neurological diseases. Some of these signs are progressive weakness; bedbound state; sleepiness; decreased intake of food and fluid; darkened and/or decreased urine output; dysphagia; delirium; decreased level of consciousness; noisy respiration/excessive respiratory tract secretion; dyspnea; change in breathing pattern (Cheyne–Stokes respiration and periods of apnea); and mottling and cooling extremities [16, 17, 27, 28]. Respiratory signs and symptoms including respiratory congestion, Cheyne–Stokes breathing, and dyspnea can induce distress and anxiety for patients, their bedside relatives, and healthcare professionals [29, 30, 31, 32]. Therefore, recognizing and addressing the signs and symptoms of imminent death in the terminal phase of life is important, not only to enhance patient comfort and ensure quality of care but also to support the well-being of relatives and curb stress in healthcare professionals [33].

Patients with neurological diseases often receive brief EOL care, even after extended hospital stays [15, 34, 35, 36, 37, 38]. Our previous study on EOL decision-making revealed that EOL decisions were made more quickly after admission to the hospital in patients with acute neurological conditions such as hemorrhagic and ischemic strokes (median: 4 days) than in those with progressive diseases like amyotrophic lateral sclerosis (ALS) and Parkinson’s disease or extrapyramidal and movement disorders (PDoed) (median: 9 and 22 days, respectively). The findings furthermore suggested that EOL care decisions were influenced by age and stroke diagnosis and not by treatment directives or demographic factors [24]. Several triggers of imminent death may occur in patients with neurological diseases several months prior to their death. These include physical deterioration, dysphagia, severe complex symptoms and pain, weight loss, respiratory issues, recurrent infections, and cognitive decline [39, 40, 41, 42, 43, 44, 45]. Understanding these signs and symptoms requires deep knowledge of the progression and symptom patterns specific to neurological diseases [43, 44, 45, 46, 47]. When using triggers to identify approaching death, careful attention must be given to the trajectory of the disease, which varies between patients and conditions [1, 9, 47, 48, 49, 50, 51]. For instance, being immobile, dependent, or bedbound are often considered general indicators of imminent death [52], but in patients with ALS, these may not signal death because reduced functional capacity can exist for long periods while patients may maintain independence in talking and eating until just hours before death [48, 52]. Conversely, patients with stroke can be paralyzed and immobile after the stroke but may enjoy fruitful and meaningful lives without actively dying [53]. Consciousness level and communication capacity are frequently assessed to monitor neurological disease progression. Changes in these signs may indicate the need to transit patients to EOL care [5, 16, 54]. However, these signs are not always reliable predictors of imminent death [5] because they can fluctuate owing to factors such as underlying medical conditions and medication use.

The need for clear guidelines for assessing signs and symptoms of imminent death has been highlighted [55]. Specific signs indicative of imminent death within 3 days have been identified in prospective studies of patients with cancer [12, 13, 16, 56]. They include neurocognitive indicators of reduced responsiveness to visual and verbal stimuli and nonreactive pupils; neuromuscular signs, such as drooping of the nasolabial folds, hyperextension of the neck, inability to close the eyelids, and grunting of the vocal cords; and neurological signs, including Cheyne–Stokes breathing, respiration with mandibular movement, and death rattle [12, 13, 16, 56]. The usefulness of these specific signs in caring for patients with neurological diseases warrants examination.

Nurses are expected to contribute to the EOL care decision-making process through their clinical assessments and professional knowledge [17, 57, 58, 59]. However, they may face challenges, including difficulty voicing concerns about ineffective treatments, balancing care needs when families pursue alternative approaches, and managing negative reactions from physicians when advocating for their patients [59]. We aimed to describe nursing contribution to EOL care decision-making and compare the signs and symptoms of imminent death in patients with acute versus progressive neurological diseases during the final 3 to 7 days of life.

## Methods

### Aim, design, and setting

We aimed to describe nursing contribution to EOL care decision-making and compare the signs and symptoms of imminent death in patients with acute versus progressive neurological diseases during the final 3 to 7 days of life. The study is a single-center, retrospective study of PHRs of documentation of EOL care decision-making, signs and symptoms indicative of impending death, and communication about EOL care with patients admitted to an acute neurological ward and their relatives in the last 3 to 7 days of life.

The study was conducted at Landspitali - University Hospital of Iceland. This hospital has the country’s only specialized acute neurological ward (population around 382,000). All nurses in the neurological ward hold a Bachelor of Science in Nursing (B.S.), and the patient-to-nurse ratio ranges from five to seven patients per nurse. The nurses report to the head nurse of the ward, who is by law accountable for nursing care provision.

### Participants

PHRs were included if they fulfilled the following criteria: Healthcare records of patients 18 years or older, admitted to the neurological ward and having the following ICD-10 codes: I60-I64 (stroke diagnoses), G12.2 (ALS), and G20 (Parkinson’s disease) or G25 (extrapyramidal and movement disorders). PHRs of patients with other diagnoses and those of patients with brief hospital stays or minimal documentation in their health records were excluded.

### Data collection

Data were collected from 271 PHRs from January 1, 2011 to August 31, 2020. The final analysis included 209 PHRs. Nurses and physicians documented signs and symptoms of impending death. Additionally, data about pain and the nurses’ role in EOL care decision-making were collected from January 1, 2017 to August 31, 2020. The inter-rater reliability of the data collection was acceptable [60].

### Instrument

The authors had previously developed a data collection tool, the Neurological End-Of-Life Care Assessment Tool, to extract data from PHRs, including (a) healthcare professionals’ contribution to EOL care (6 items) with identification of patients’ health status as progressing (documented as a worsening or deteriorating condition of the patient) and/or needing EOL care (documented as the need for a treatment directive of EOL care), (b) documentation of EOL care decision-making written by the responsible physician (1 item), (c) signs and symptoms that may inform impending death (22 items), and (d) communication with relatives (3 items) [59]. All items in the Neurological-End-Of-Life Assessment Tool have a nominal value. Additionally, we obtained data about nurses’ contribution to EOL care decision-making from January 1, 2017 to August 31, 2020, using the following three items identified in the RNAO guidelines [17] and relevant literature [21, 23]: information broker, advocate, and supporter. Information broker refers to nurses providing clear, timely information to help patients and families understand treatment options and decisions. Supporter denotes nurses offering emotional and practical support to help patients and families cope with end-of-life challenges, and advocate connotes nurses ensuring that patients’ wishes are respected and upheld in the decision-making process [23]. Each of the three items is reflected in three statements, totaling nine statements, which all have nominal value.

### Statistical analysis

All statistical analyses were conducted using the IBM Statistical Package for Social Sciences for Windows (Version 29.0; IBM Corp., Armonk, N.Y., USA). We used descriptive statistical methods to summarize categorical variables, presenting them as frequencies and percentages, and used inferential statistical methods to compare groups. We used the t-test to identify differences in the frequency of signs and symptoms. The chi-square test was used to determine differences in the frequencies of imminent death signs and symptoms between patients receiving and not receiving EOL care, as well as between patients with acute and progressive neurological diseases. Statistical significance was set at *p* < 0.05. To provide an in depth understanding of nurses’ decision-making beyond the statistical results, excerpts from the PHRs were extracted to illustrate how nurses document patients’ need to transition to EOL care.

### Ethics approval

This study was conducted in accordance with the declaration of Helsinki. The study obtained approval from the Ethics Committee at Landspitali, University Hospital of Iceland (reference number 26/2017) for extracting data from PHRs from January 1, 2011 to August 31, 2020. It was reported to the Icelandic Data Protection Authority. A renewal was granted in July 2023 (Nr. 36/2023). This study uses coded data extracted from PHRs, hence no direct patient contact or patient identifiable patient data. Therefore, informed consent was waived by the Ethics committee at Landspitali University Hospital of Iceland and deemed unnecessary according to National Regulations.

## Results

### Recognition of impending death

Progression of the patient’s disease was recognized in a total of 96% of cases, with nurses and physicians documenting deterioration in 44% and 79% of cases, respectively. Documentation was complete (100%) across all patient groups, except for patients with ischemic stroke, where it was 90% (Table 1). The need for EOL care was documented in 85% of cases, with 36% and 76% documentation rates for nurses and physicians, respectively. The highest frequency of documentation of the need for EOL care by nurses was for patients with ALS (48%), while the lowest was for those with PDoed (9%).

**Table 1:** Disease progression and need for end-of-life care (*N* = 209)

	Total (*N* = 209)	Stroke– ischemic (*n* = 123)	Stroke– hemorrhagic (*n* = 48)	ALS* (*n* = 27)	PDoed** (*n* = 11)
	*N* (%)	*n* (%)	*n* (%)	*n* (%)	*n* (%)
**Identified progression of disease**	196 (94)	111 (90)	48 (100)	27 (100)	11 (100)
Nurses	92 (44)	52 (42)	22 (46)	16 (59)	2 (18)
Physicians	165 (79)	93 (76)	43 (90)	21 (78)	8 (73)
Identified need for EOL*** care	178 (85)	97 (79)	40 (83)	23 (85)	8 (73)
Nurses	75 (36)	45 (37)	16 (33)	13 (48)	1 (9)
Physicians	158 (76)	90 (73)	41 (85)	19 (70)	8 (73)

*ALS, amyotrophic lateral sclerosis; **PDoed, Parkinson’s disease or extrapyramidal and movement disorders; ***EOL, end of life

Table 2 shows three distinct roles of nursing contribution to EOL care decision-making. The contribution was most frequent in the role of information broker, with the most frequent individual items being signs and symptoms of impending death (19%) and the need for EOL care (13%).

**Table 2:** Nursing contribution to decision-making on end-of-life care (*N* = 85)

Categories	Total	
	*N* (%)	*n* (%)
**Information broker**	36 (15)	
End-of-life care is needed		11 (13)
Signs and symptoms of impending death		17 (19)
Communicated with relatives about deterioration		8 (9)
**Advocate**	**12 (6)**	
Contacted physician about the need for end-of-life care		5 (6)
Arranged for a family meeting		6 (7)
Identified need for contacting palliative care team		1 (1)
**Supporter**	**14 (6)**	
Suggested end-of-life care		6 (7)
Allowed family time to process information		4 (5)
Informed relatives about deterioration		4 (5)

Table 3 shows excerpts (free text) from the PHRs on how nurses argued for EOL care. These statements reflect clinical judgement of critical symptoms such as unconsciousness, irregular pupil reactions, and rapid breathing to signify the severity of the patient’s condition, communication with the patient’s family, prompt notification of a spouse about the deteriorating condition, and recognition of urgent family’s needs, as well as facilitation of expedient medical care.

**Table 3:** Excerpts showing argumentation for the need for transition to end-of-life care

Patient with stroke	“During handover, the patient was unconscious and unresponsive to verbal and painful stimuli. The right pupil was fixed and dilated, and the left was near pinpoint. Neither pupil reacted to light. Breathing was rapid but steady. An acute CT** scan revealed a large infarction in the right hemisphere with herniation, indicating imminent death. The patient’s wife was called and informed of the critical situation and was advised to come as quickly as possible.”
Patient with stroke	“The patient seems to be close to dying, but the family is struggling to accept how advanced the disease is. It would be helpful if the physician talks with them again tomorrow and considers adding midazolam PRN***, alongside diazepam and morphine. The dyspnea episodes were difficult for the patient last night; it would have been better for her to receive midazolam rather than repeated suctioning. The patients’ (adult) children do not realize the severity of her condition.”
Patient with ALS*	“It was a difficult night after 3 a.m. The patient received intravenous midazolam, morphine, and diazepam. A decision is needed regarding possible transition to end-of-life care and ensuring comfort in the patient’s final days. He has received tube feeding, but it was stopped today. The patient was awake from 12–5 pm and felt awful; he was grimacing and making sounds. At 4.30 p.m. the patient received 10 mg morphine subcutaneously PRN. It was tough for the family to accept that he was dying, especially seeing him in distress. It is important to reevaluate the current care approach, including whether end-of-life treatment would be appropriate so that adequate sedation could be provided to prevent him from being awake in such discomfort. When he wakes up, he is really bad, he gets all worked up, so it takes time for him to relax again.”
Patient with Parkinson’s disease	“A family meeting has been scheduled for 1 p.m. tomorrow where they will probably decide on end-of-life care. The patient is currently only receiving fluids through a feeding tube. A physician has already reported the deterioration in the patient’s condition to the patient’s grandchildren.”

*ALS, amyotrophic lateral sclerosis; **CT, computed tomography; ***PRN, pro re nata

### Demographics of patients

Table 4 presents the characteristics of 209 patients diagnosed with ischemic stroke (*n* = 123), hemorrhagic stroke (*n* = 48), ALS (*n* = 27), or PDoed (*n* = 11). The average age at the time of death was 79 years (standard deviation = 11.3), with 51% of the patients being women and 55% being married or cohabiting.

**Table 4:** Demographic characteristics of the analyzed patients (*N* = 209)

	Total (*N* = 209)	Stroke– ischemic (*n* = 123)	Stroke– hemorrhagic (*n* = 48)	ALS* (*n* = 27)	PDoed** (*n* = 11)
	*n* (%)	*n* (%)	*n* (%)	*n* (%)	*n* (%)
Age at death (years)					
30–49	4	2	2	0	0
50–69	33	12	6	11	4
70–89	144	84	37	16	7
90+	28	25	3	0	0
Women	106 (51)	70 (57)	21 (44)	12 (44)	3 (27)
Men	103 (49)	53 (43)	27 (56)	15 (56)	8 (73)
Single/widowed/divorced	86 (45)	59 (54)	17 (40)	9 (35)	1 (9)
Married/cohabiting	104 (55)	51 (46)	26 (60)	15 (65)	10 (91)
Treatment directive for end-of-life care	181 (87)	108 (88)	44 (92)	21 (78)	8 (73)

*ALS, amyotrophic lateral sclerosis; **PDoed, Parkinson’s disease or extrapyramidal and movement disorders

### Comparison and frequency of signs and symptoms between patient groups

Overall, 87% (181) of the patients had a documented EOL care decision. Of the 22 signs and symptoms of imminent death, 5 were excluded owing to a documentation frequency less than 10%, leaving 17 signs and symptoms for further analysis. The remaining signs and symptoms were Cheyne–Stokes breathing, decreased urine output, respiratory congestion, decreased response to verbal stimuli, decreased response to visual stimuli, non-reactive pupils, grunting of vocal cords, no food intake, no fluid intake, fatigue, nausea, dyspnea, agitation, pain, unconscious/limited consciousness, communication (not-responsive), and being bedbound.

The mean number of signs and symptoms in the entire study cohort was 6.5 (range: 1 to 14). The difference in the mean number of signs and symptoms between patients with and without EOL care decision was not significant in the ischemic stroke (6.5 vs. 6.7, *t* = − 0.378, *df* = 121, *p =* 0.706), ALS (5.9 vs. 5.8, *t* = 0.070, *df* = 25, *p* = 0.945), and PDoed groups (5.8 vs. 5.3, (*t* = 0.336, *df* = 9, *p* = 0.744); however, a significant difference was noted in the hemorrhagic stroke group, regardless of the assumption of equal variances (7.2 vs. 4.0, *t* = 2.246, *df* = 46, *p* = 0.030).

**Fig. 1:**
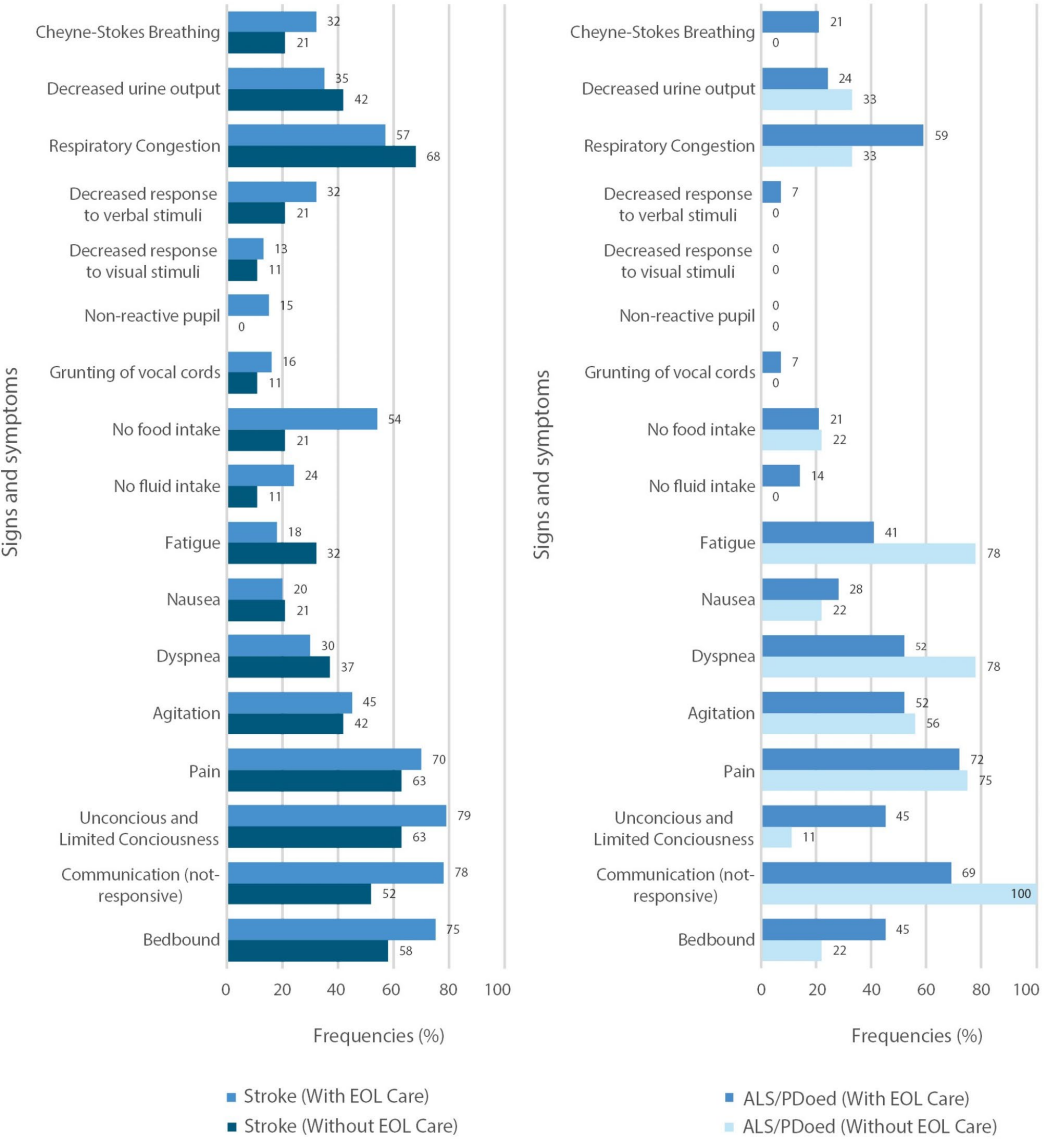
Comparison of the frequencies of documentation of signs and symptoms of imminent death

Figure 1 shows the difference in the frequency of signs and symptoms between those with and without EOL care decision for all patient groups. For patients with stroke (hemorrhagic and ischemic with EOL care decision, the most common signs and symptoms were unconsciousness or limited consciousness (79%), a non-responsive communication status (78%), immobility or being bedbound (75%), pain (70%), and no food intake (54%). Patients with stroke with EOL care decision had lower frequencies of respiratory congestion (57%), decreased urine output (35%), dyspnea (30%), nausea (20%), and fatigue (18%) than those without EOL care decision.

Among patients with ALS or PDoed, those with EOL care decision had higher frequencies of respiratory congestion (59%), unconsciousness or limited consciousness (45%), being bedbound (45%), nausea (28%), and Cheyne–Stokes breathing (21%) than those without EOL care decision, see Fig. 1. However, they had lower frequencies of pain (72%), a non-responsive communication status (69%), dyspnea (52%), agitation (52%), fatigue (41%), decreased urine output (24%), and no food intake (21%) than patients without EOL care decision.

To further analyze group differences of patients who had documented EOL care decision, significant differences were found among the patient groups. Patients with EOL care decision more commonly had no food intake (*chi-square* = 7.24, *df* = 1, *p =* 0.007) and fatigue (*chi-square* = 8.03, *df* = 1 *p =* 0.005) than those without EOL care decision. Among patients with stroke, a non-responsive communication status was significantly more frequent among those with EOL care decision than in those without EOL care decision (78 vs. 52, *chi-square* = 6.00, *df* = 1, *p =* 0.014). While not statistically significant, patients with ALS/PDoed without EOL care decision showed a higher frequency of a non-responsive communication status than those with EOL care decision (100% vs. 69%, *chi-square* = 3.660 *df* = 1, *p* = 0.056). Additionally, non-significant trends emerged, showing higher frequencies of non-reactive pupils (15% vs. 0%, *chi-square* = 3.156, *df* = 1 *p =* 0.076) in patients with stroke with EOL care decision compared to those patients with stroke without EOL care decision. In patients with ALS/PDoed, unconsciousness or limited consciousness was more frequent among those with EOL care decision than in those without EOL care decision (45% vs. 11%, *chi-square* = 3.356, df = 1, *p =* 0.067).

**Fig. 2:**
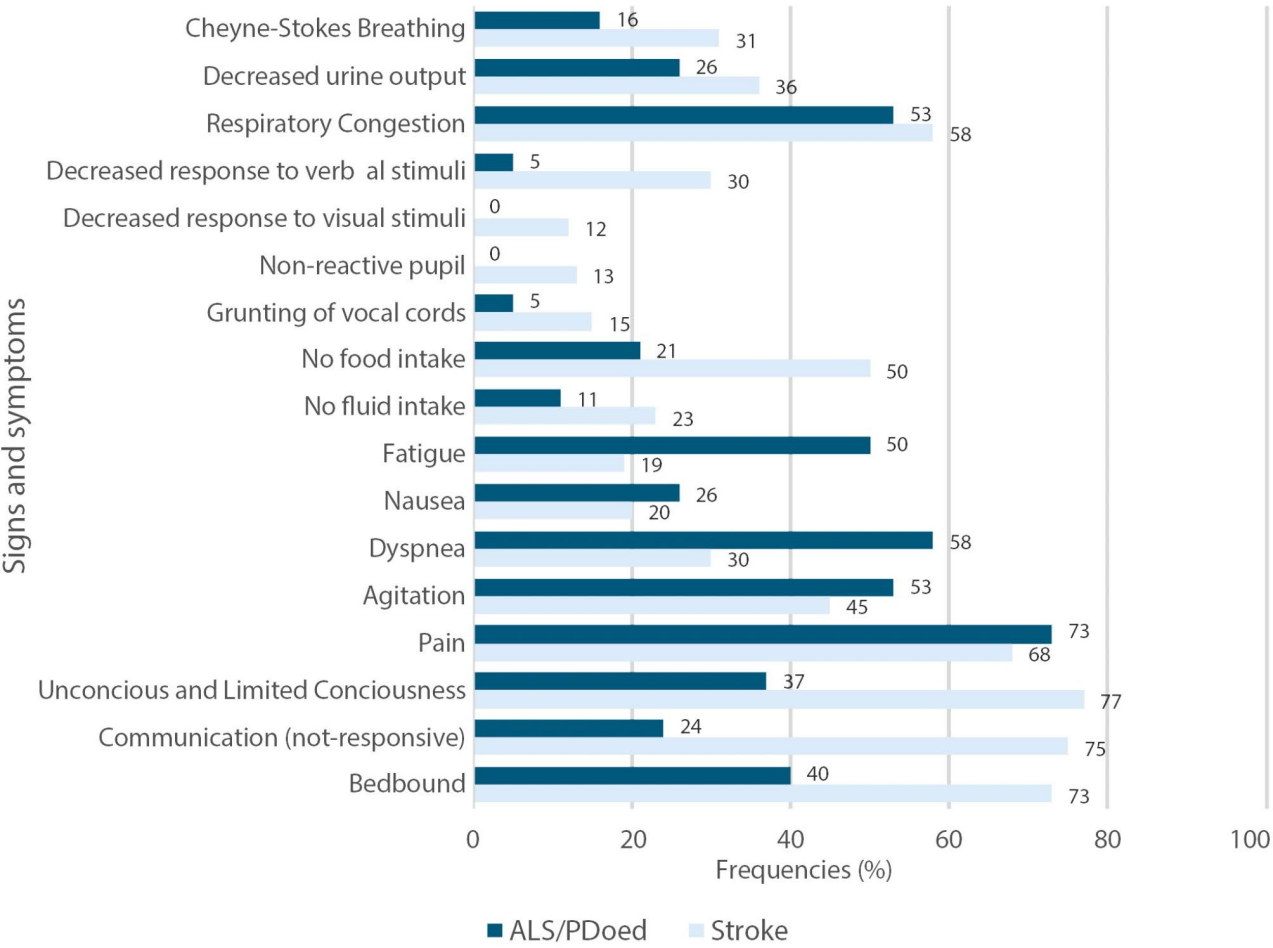
Frequencies of signs and symptoms of patients according to disease category

Figure 2 shows the distinct pattern that emerged when we grouped patients based on EOL care decision —acute (ischemic and hemorrhagic stroke) versus progressive (ALS and PDoed). The most pronounced differences were noted in a non-responsive communication status (75%, *chi-square* = 37.13, *df* = 1, *p =* 0.001), being bedbound (73%, *chi-square* = 15.90, *df* = 1, *p =* 0001), no food intake (50%, *chi-square* = 10.74, *df* = 1, *p =* 0.001), unconsciousness or limited consciousness (77%, *chi-square* = 24.04, *df* = 1, *p =* 0.001), and non-reactive pupils (13%, *chi-square* = 5.46, *df* = 1, *p =* 0.019), all of which were more common in patients with stroke than in patients with ALS or PDoed. Conversely, dyspnea (58%, *chi-square* = 10.27, *df* = 1, *p =* 0.001) and fatigue (50%, *chi-square* = 15.68, *df* = 1, *p =* 0.001) were more frequently documented in patients with ALS or PDoed than in those with stroke.

### Relatives and family meetings

Table 5 shows that the frequency of informal conversations about EOL care decision-making with patients ranged from 4 to 63%, with the most frequent conversations being documented in those patients with ALS. The frequency of informal conversations with the relatives ranged from 73% (Patients with PDoed) to 87% (Patients with ischemic stroke), and formal family meetings were conducted in most cases or in 82% (Patients with PDoed) to 96% (Patients with hemorrhagic stroke) of cases in the final week of life.

**Table 5:** Conversations of health care professionals with relatives about end-of-life care (*n* = 209)

	Total (*N* = 209)	Stroke– ischemic (*n* = 123)	Stroke– hemorrhagic (*n* = 48)	ALS* (*n* = 27)	PDoed** (*n* = 11)
	*N* (%)	*n* (%)	*n* (%)	*n* (%)	*n* (%)
Informal conversation on EOL*** care with patient	29 (14)	9 (7)	2 (4)	17 (63)	1 (9)
Informal conversation on EOL care with relatives	178 (85)	107 (87)	40 (83)	23 (85)	8 (73)
Family meeting	194 (93)	116 (94)	46 (96)	23 (85)	9 (82)

*ALS, amyotrophic lateral sclerosis; **PDoed, Parkinson’s disease or extrapyramidal and movement disorders; ***EOL, end of life

## Discussion

In this study, we examined nursing contributions to EOL care decision-making in patients with neurological diseases, recognition of signs and symptoms of impending death, and interactions with patients and relatives. These aspects are inherently linked to the ultimate EOL care decision-making ordered by physicians. The findings demonstrate that nurses played a multifaceted and essential role, which resonates with recommendations in the literature [17, 56, 57]. The majority of the patients had a documented EOL care decision. Essential to EOL care decision-making is recognizing signs and symptoms of impending death. The mean number of signs and symptoms in all groups (ischemic and hemorrhagic stroke, ALS, and PDoed), was 6.5 and ranged from 1 to 14 in the 3–7 days leading up to death, with patients with hemorrhagic stroke having the highest frequency of documented symptoms (7.2). This is consistent with findings from other studies [3, 4, 8, 10, 15, 16]. The existence of multiple signals should alert health professionals to the approaching EOL. In patients with ALS or PDoed, the documentation focused on dyspnea, agitation, and fatigue, reflecting the progressive nature of their conditions. Conversely, documentation of patients with stroke highlighted issues related to lowered consciousness state, being bedbound, and lack of food intake, underscoring neurological impairment and paralysis. Notably, both groups shared significant overlaps in having a non-responsive communication status, pain, and respiratory congestion, illustrating common challenges these patients face.

Hui et al. [16, 54] conducted prospective studies on signs of imminent death, with a predefined protocol for nurses on how to observe and document these signs. In our study, out of 12 of those signs, 7 had acceptable documentation and were used to report signs of imminent death, namely respiratory congestion, Cheyne–Stokes breathing, decreased response to verbal and visual stimuli, grunting of vocal cords, and non-reactive pupils. The remaining five signs—respiration with mandibular movement, pulselessness of the radial artery, inability to close the eyelids, hyperextension of the neck, and drooping of the nasolabial fold—were documented in less than 10% of the cases. A key difference between our retrospective study and others [12, 13, 16] is that we did not predefine specific factors for healthcare professionals to observe in the clinical setting, allowing us to capture the issues that the healthcare professionals considered needing documentation. Such insight is of value to inform this complicated practice, something that is difficult to depict with prospective study designs.

Patients with stroke, especially those with hemorrhagic stroke, had a higher frequency of documented symptoms than those with ALS or PDoed, likely owing to the acute nature of stroke. Stroke symptoms typically appear suddenly and require close surveillance in the acute phase [36, 38], which leads to more detailed clinical documentation. In contrast, ALS and PDoed symptoms progress gradually [8, 15], potentially resulting in less frequent or detailed documentation. Nurses tend to focus on urgent issues, such as changes in consciousness in patients with stroke, while slower changes in ALS or PDoed, such as reduced response to verbal and visual stimuli, receive less emphasis. This may explain the more thorough documentation in patients with stroke. Further exploration of these findings is needed, but enhancing education on signs and symptoms of approaching death and suggesting preliminary criteria for symptom monitoring may be a beneficial first step toward improving documentation [1].

The findings on communication with patients and relatives reveal differences across different disease groups (ischemic and hemorrhagic stroke, ALS, and PDoed), suggesting variations in their involvement in care decisions. Informal conversations with patients were more common for progressive diseases like ALS than for hemorrhagic stroke. Patients with ALS were also more likely to be conscious and communicating during the final days, while communication with patients with hemorrhagic stroke was minimal. A Swedish study using national EOL registry data found that 93% (*n* = 770) of patients with ALS were still communicating, and 73% (*n* = 603) had EOL care discussions with healthcare professionals [11], which is considerably higher than that in our study. A lack of communication guidelines with ALS patients has been identified [14]. Improved competencies among health professionals are needed, encouraging early EOL care discussions with patients facing progressive neurological diseases.

The findings of this study indicate that conversations and formal meetings with relatives of patients about the condition of patients were common during the final week of life. Relatives often provide key insights into patients’ values and wishes, enabling healthcare professionals to tailor care to meet individual needs better. When patients are unconscious or unable to make their own decision, the importance of the relatives in the decision-making process increases. Similar to our study, Ozanne et al. [4] reported a 69–89% prevalence of EOL care discussions with family members, highlighting the beneficial effects of such communication. Similarly, Erikson et al. [10] found that the families of 74% (*n* = 1195) of patients with stroke received informative communication about transitioning to EOL care. At the same time, Eljas Ahlberg et al. [11] reported that 81% (*n* = 671) of patients with ALS had transitioned to EOL care discussions.

Evidence-based clinical guidelines on EOL care for patients with neurological diseases are emerging (44,45). Although in their early stages of development, differentiating between the needs of patients with acute (44) and progressive (45,53) neurological diseases has started to take shape, which is congruent with the main implications of this study. Early identification of patients with neurological diseases needing EOL care is a priority, concurrent with further development of evidence-based clinical guidelines for such care. The findings of this study may be useful for that purpose. At the same time, it is crucial to explore further the factors that may contribute to delayed recognition of approaching death in acute care settings [1, 3]. This includes assessing whether signs and symptoms of imminent death are mistaken for acute reversible symptoms [1].

### Strengths and limitations

Using real-world data from a specialized neurological ward strengthens the relevance of this study’s findings. However, owing to its retrospective nature, existing documentation was used, which may overlook certain care details and affect the results. While reflective of everyday practice, the single-center setting limits the generalizability of the findings to other healthcare environments. This study assessed various signs and symptoms, comparing those documented in patients with and without EOL care decisions and between individuals with acute and chronic neurological diseases. These comparisons should be interpreted cautiously due to variability documentation and the overall low frequency of documentation. Despite these limitations, the study offers valuable insights into EOL care decision-making in neurological settings, and indicates trends in difference between patient groups, which can inform nurses’ training, improve documentation, and enhance interdisciplinary communication, ultimately leading to better patient care.

## Conclusions

Nurses made substantial contributions to EOL care decision-making for patients with neurological diseases, for which the majority had a documented decision. The frequency of signs and symptoms of imminent death was similar between those with and without EOL care decision, except for patients with hemorrhagic stroke, who exhibited a higher frequency among those with EOL care decision compared with those without EOL care decision. The symptom burden was notable for patients without EOL care decision, particularly those with progressive diseases. Further exploration of factors contributing to delayed recognition of the need for EOL care decision in acute settings is paramount. This includes assessing whether the signs and symptoms of imminent death are clear to healthcare professionals or are misinterpreted as acute, reversible conditions.

## Data Availability

The data that supports the findings of this study are available from the corresponding author upon reasonable request.

## Abbreviations

ALS: Amyotrophic lateral sclerosis
EOL: End of life
PHRs: Patient health records
PDoed: Parkinson’s disease or extrapyramidal and movement disorders
RNAO: Registered nurses association of Ontario
